# Repeated decrease of CD4+ T-cell counts in patients with rheumatoid arthritis over multiple cycles of rituximab treatment

**DOI:** 10.1186/s13075-016-1152-5

**Published:** 2016-10-28

**Authors:** Matthieu Lavielle, Denis Mulleman, Philippe Goupille, Clément Bahuaud, Hsueh Cheng Sung, Hervé Watier, Gilles Thibault

**Affiliations:** 1 Université François-Rabelais de Tours, CNRS, UMR 7292, Tours, France; 2 Service de Rhumatologie, CHRU de Tours, Tours, France; 3 Laboratoire d’Immunologie, CHRU de Tours, Tours, France; 4 GICC – UMR 7292, UFR de Médecine, Bâtiment Vialle, 10 boulevard Tonnellé, BP 3223, 37032 Tours, Cedex 01 France

**Keywords:** Rheumatoid arthritis, Rituximab, CD4+ T cells, Biomarker

## Abstract

**Background:**

Significant peripheral blood CD4+ T-cell depletion has been observed after a first cycle of rituximab, a monoclonal antibody directed against the CD20 antigen, which is currently used in rheumatoid arthritis. Of note, an absence of CD4+ T-cell decrease has been observed in non-responders. Herein, we describe CD4+ T-cell changes over repeated cycles of rituximab and their relationship with clinical outcomes.

**Methods:**

Patients with rheumatoid arthritis who started rituximab between July 2007 and July 2013 were analyzed up to November 2014. Lymphocyte phenotyping and clinical assessments were performed before, and 3 and 6 months after each cycle. Lymphocytes counts and disease activity were compared at each time point, using nonparametric tests.

**Results:**

Patients received up to seven cycles of treatment during the study period. Mean CD4+ T-cell counts were above the upper limit of the reference range before each rituximab infusion and repeatedly reached the reference range at 6 months (and/or 3 months) post infusion. CD4+ T cells decreased concurrently with disease activity score.

**Conclusions:**

CD4+ T-cell counts could be a relevant biomarker of response to rituximab in rheumatoid arthritis and could be considered in making decisions about the timing of retreatment.

## Background

Targeting B cells with rituximab (RTX), a chimeric monoclonal antibody directed against the CD20 antigen, induces a sustained clinical improvement in most patients with rheumatoid arthritis (RA) [1, 2]. Patients who respond to treatment are likely to receive multiple repeated cycles of RTX upon relapse.

RTX leads to depletion of both normal and autoreactive B cells and this effect is almost complete in blood and partial in bone marrow [3–7]. The latter population is responsible for the production of rheumatoid factor (RF) and anti-citrullinated protein antibodies (ACPAs), both hallmarks of RA. Although some studies have shown that RF [8, 9] or ACPA [8] positivity are associated with clinical response, RTX is also effective in the RF-negative and ACPA-negative forms of RA. Additionally, Cambridge et al*.* observed a post-RTX decrease in RF and ACPA but neither the pre-treatment levels nor the changes observed after treatment were predictive of response to treatment [10]. These data suggest that the efficacy of RTX in RA may depend on B-cell functions unrelated to autoantibody production.

We and others previously reported significant depletion of CD4+ T cells in peripheral blood in patients receiving a first cycle of RTX for treatment of RA [11, 12]. Interestingly, patients who were non-responders to this first cycle tended to experience a milder depletion of CD4+ T cells than responders, whereas depletion of B cells was similar in both groups. However, the relationship between changes in CD4+ T cells and clinical outcomes over repeated cycles of RTX has not been studied. The aim of the present study was to describe and characterize the changes in CD4+ T cells over multiple, repeated cycles of RTX and to investigate the potential relationship between these changes and disease activity.

## Methods

### Patients and study protocol

Patients seen in routine clinical practice, who started RTX for the treatment of RA between July 2007 and July 2013, were included in this retrospective study. The present study is based on data collected during follow up to November 2014. Therefore, some patients included at the end were not followed up for as long as those who were recruited at the beginning. Patients received two infusions of 1000 mg of RTX as previously described. Clinical improvement (measured by the Disease Activity Score in 28 joints using erythrocyte sedimentation rate (DAS 28-ESR) and clinical tolerance were assessed 3 months (M3) and 6 months (M6) post infusion [11]. Radiographs were available at baseline, but radiographic progression was not assessed systematically. Per-cycle clinical response using European League Against Rheumatism (EULAR) criteria was calculated for each patient relative to the baseline DAS 28-ESR of the first cycle [13]. Decisions about retreatment and treatment intervals were based on clinical response to the previous cycle and symptoms of relapse after M6 (as-needed basis). Lymphocyte phenotyping was carried out before each infusion and at follow-up visits as per routine procedure, without additional sampling.

### Lymphocyte phenotyping by flow cytometry

Lymphocyte phenotyping was performed as previously described according to a standard no-wash, whole-blood procedure using a PrepPlus and a TQ-Prep workstation or a FP1000 workstation (Beckman Coulter) and an Epics XL-MCL or a Navios flow cytometer (Beckman Coulter) [11].

### Statistical analysis

Statistical analysis was performed using GraphPad Prism® software (version 6.0 for Macintosh; GraphPad Software, San Diego, CA, USA). Wilcoxon’s matched-pairs signed rank test was used for analysis of paired data and differences in continuous variables between non-paired data were assessed using the Mann–Whitney nonparametric test. Results are presented as median and range (minimum (min)-maximum (max)) or interquartile range (IQR) for continuous variables. Kaplan-Meier curves were used to study the persistence of patients under rituximab. The significance level was set at 5 % (*p* < 0.05).

## Results

### Mean global disease activity, CD19+ B-cell and CD4+ T-cell counts over multiple cycles of RTX

**Fig. 1:**
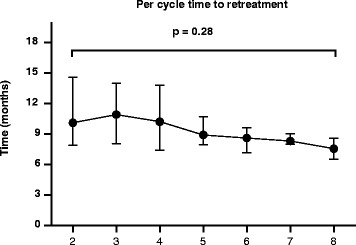
Time interval between each rituximab cycle. Results are presented as median and interquartile range

**Fig. 2:**
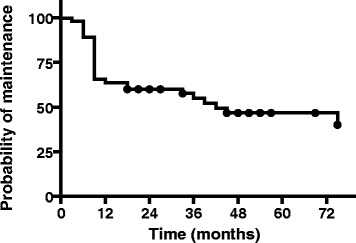
Probability of rituximab maintenance versus time in 54 patients with rheumatoid arthritis

**Fig. 3:**
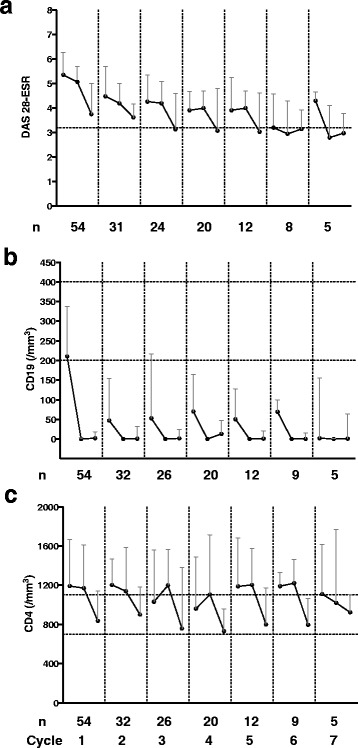
Changes in Disease Activity Score in 28 joints-erythrocyte sedimentation rate (*DAS28-ESR*) (**a**), CD19 + B cells (**b**) and CD4+ T cells (**c**) over seven cycles of rituximab (RTX). For each cycle, values are presented at three time points, i.e. before the first infusion, before the second infusions two weeks apart and at 3 or 6 months post treatment. Results are shown as the median and interquartile range in patients with available data. The level defining low disease activity is represented (DAS28 ≤ 3.2). The limits of the reference range are represented for CD19+ B cells (between 200 and 400/mm^3^) and for CD4+ T cells (between 700 and 1100/mm^3^)

Fifty-four patients started RTX during the study period. Patients’ characteristics are presented in Table 1. The time interval between each cycle is presented in Fig. 1. Maintenance to treatment is presented in Fig. 2. Median time to RTX discontinuation was 42 months. Adverse events were responsible for discontinuation in 4 of the 54 patients after the first cycle. Changes in disease activity and lymphocyte counts over repeated cycles of RTX are presented in Fig. 3. For each cycle, values are presented at three time points, i.e. before the first infusion, before the second infusions two weeks apart and at 3 or 6 months thereafter. All but one patient received methylprednisolone.

**Table 1:** Baseline characteristics of the 54 patients

Characteristics	Baseline value
Age, years	60 (36–84)
Sex, *n* (%) female	42 (78)
Disease status	
Disease duration, years	16 (1–36)
Disease Activity Score in 28 joints-erythrocyte sedimentation rate	5.3 (2.1–7.7)
Erythrocyte sedimentation rate, mm/h	33.4 (3.0–111.0)
C-reactive protein, mg/L	17 (1–166)
Rheumatoid factor positivity, *n* (%)	38 (70)
Anti-cyclic citrullinated peptide positivity, *n* (%)	47 (87)
Radiologic evidence of erosions, *n* (%)	42 (78)
Previous treatment, *n* (%)	
Anti-tumor necrosis factor-α	42 (78)
Methotrexate	24 (52)
Prednisone	42 (78)
Lymphocyte immunophenotype	
CD19+ cells/mm3	211 (25–706)
CD3+ cells/mm3	1,740 (323–3,378)
CD4+ cells/mm3	1,192 (233–2,882)
CD8+ cells/mm3	482 (120–1,114)

Values are the median (min–max) unless stated otherwise

Repeated RTX treatment led to sustained clinical improvement with the mean post-treatment DAS28-ESR tending to decrease with the number of cycles (Fig. 3a). Clinical improvement was delayed with B-cell depletion, the latter being achieved before the second infusion in most patients (Fig. 3b). Whilst the cycle 1 pre-treatment B-cell count was within the normal reference range, it remained substantially below the lower limit of the reference range (<200/mm^3^) thereafter, and tended to decrease with the number of cycles. Hence, patients were usually retreated before recovery of the circulating B-cell pool.

The changes occurring in CD4+ T-cell counts were somewhat different (Fig. 3c). First, mean CD4+ T-cell counts before RTX infusions were systematically above the upper limit of the reference range (>1100/mm^3^). They were higher in women than in men (*p* < 0.01), in patients without RF than in those with RF (*p* < 0.04), in patients without radiologic evidence of erosion than in those with evidence of erosion (*p* < 0.03) and in RTX first-cycle responders than in non-responders (*p* < 0.03). Of note, there was a trend towards higher pre-treatment CD4^+^ T-cell counts in patients previously treated with TNF inhibitors as compared to patients not previously treated (*p* < 0.09). Second, the mean post-treatment counts (M3 or M6) were substantially decreased (within the reference range), regardless of the cycle. The decreased CD4+ T-cell counts were independent of sex, RF and ACPA status, erosion status and co-medication with corticosteroids or methotrexate (data not shown). The decrease was delayed compared to B-cell depletion and was closely related to clinical improvement. Of note, there was no correlation between the CD4+ T-cell count and DAS28 at baseline (data not shown). Finally, CD4+ T-cell recovery was almost complete at the time of each re-treatment. Changes in DAS 28, CD19+ and CD4+ cells, according to categories of patients (i.e. men vs women, RF+ vs RF-, erosion vs non-erosion, previous TNF inhibitors vs no previous TNF inhibitors) during the first cycle are presented in Table 2 for the 32 patients who received a second cycle.

**Table 2:** DAS28, CD 19+ cell count, CD4+ cell count at baseline, post treatment (M3/M6) and at the time of retreatment during the first cycle in 32 patients who received a second cycle of rituximab

	DAS28-ESR			CD19+ (cells/mm^3^)			CD4+ (cells/mm^3^)		
	Pre-treatment	Post treatment	Retreatment	Pre-treatment	Post treatment	Retreatment	Pre-treatment	Post treatment	Retreatment
Men (n = 7)	5.4 (4.1–6.0)	4.4 (2.0–5.1)	3.8 (3.2–5.3)	180 (45–237)	9 (0–87)*	50 (3–138)*	962 (703–1047)	652 (393–853)	1146 (702–1412)
Women (n = 25)	5.2 (4.6–6.2)	3.3 (2.7–4.7)**	4.6 (3.8–5.9)**	320 (179–411)	3 (0–36)**	44 (11–171)**	1329 (1015–1873)	988 (772–1498)**	1325 (964–1549)*
RF + (n = 22)	5.3 (4.3–6.1)	3.6 (2.6–5.1)**	4.3 (3.5–5.5)	271 (124–387)	3 (0–21)**	47 (10–134)**	1041 (908–1640)	875 (603–1305)*	1033 (826–1513)
RF – (n = 10)	5.2 (4.7–6.3)	3.3 (3.1–4.6)*	4.6 (3.6–5.8)*	275 (191–386)	15 (0–55)**	45 (8–263)*	1442 (1121–2048)	1044 (864–1386)**	1376 (1212–1637)*
Erosion (n = 24)	5.8 (4.3–6.4)	3.7 (2.6–5.1)**	4.6 (3.5–5.8)*	233 (143–325)	3 (0–40)**	47 (7–131)**	1119 (953–1708)	998 (639–1514)**	1328 (927–1584)*
No erosion (n = 8)	5.0 (4.7–5.3)	3.4 (2.7–4.3)*	4.2 (3.5–5.4)	371 (147–541)	4 (1–38)	100 (22–229)*	1289 (912–2201)	939 (869–981)*	1112 (958–1345)*
TNFi (n = 27)	5.2 (4.5–6.3)	3.6 (2.7–5.0)**	4.3 (3.4–5.8)*	237 (141–332)	3 (0–30)**	44 (9–138)**	1248 (995–1732)	988 (652–1464)**	1331 (1029–1620)*
No TNFi (n = 5)	5.8 (4.4–6.0)	2.8 (2.4–5.0)	4.4 (3.9–5.0)	433 (244–523)	3 (1–256)	68 (8–360)	819 (739–1733)	691 (323–907)	918 (727–981)

Patients were grouped according to the following subgroups: men/women, rheumatoid factor (RF)+/RF-, erosion/no-erosion, previous TNF inhibitor (TNFi)/no previous TNFi. Results are presented as median and interquartile range. *DAS28-ESR* Disease Activity Score in 28 joints-erythrocyte sedimentation rate. Wilcoxon’s matched-pairs signed rank test was used to compare values post treatment vs pre-treatment, and retreatment vs post treatment: *p < 0.05, **p < 0.005

### Per-cycle variation in CD4+ T-cell counts induced by RTX

**Fig. 4:**
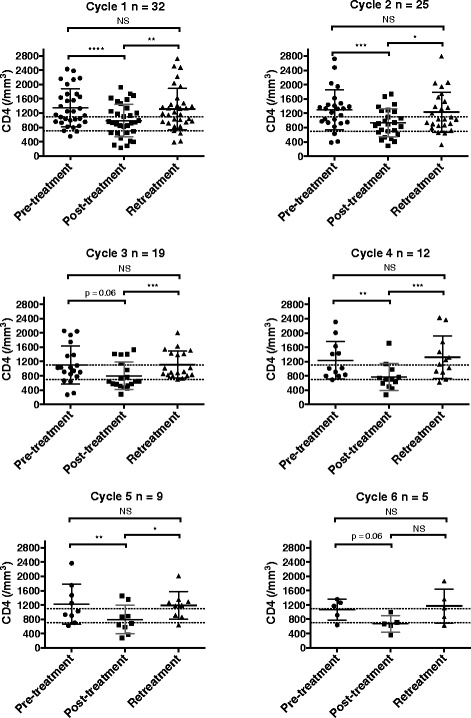
Per-cycle analysis of pre-treatment, post-treatment and retreatment counts of CD4+ T cells. In each cycle only patients for whom data were available were included. Each *dot* represents one patient and *lines* represent the mean with SD. The limits of the reference range are presented (between *700* and *1100/mm* ^*3*^). CD4+ cell counts were compared between the three points, i.e. before treatment, 3 or 6 months post treatment and before retreatment for each cycle usingWilcoxon’s matched-pairs signed rank test: ****p < 0.0001, ***p < 0.001, **p < 0.005, *p < 0.05; *NS* not significant

**Fig. 5:**
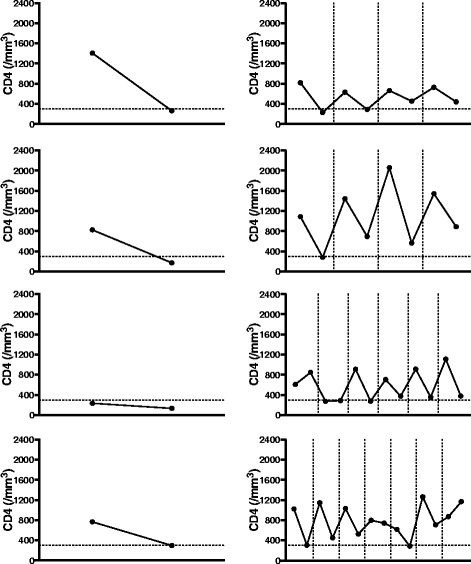
Changes in CD4+ T cell counts in patients (n = 8/54) who experienced at least one decrease in the CD4+ cell count to below 300/mm3 during follow up

To more precisely describe the dynamic changes induced by RTX, CD4+ cell counts were compared at three time points in each cycle: pre-treatment, post treatment (i.e. at M6 or at M3 if M6 data were unavailable) and retreatment (i.e. before the first infusion of the next cycle). For these per-cycle analyses, only retreated patients were included (Fig. 4) in order to describe both decrease and recovery phases. For all cycles except cycle 3 and cycle 6, mean post-treatment CD4+ values were significantly lower than mean pre-treatment or retreatment values. The mean post-treatment count tended to decrease slightly upon repeated cycles, reaching the lower limit of the reference range after cycle 4. Furthermore, RTX led to dramatically decreased post-treatment CD4+ cell counts, i.e. ≤300 cells/mm^3^ in 8/54 patients (6 patients in the first cycle), including in one patient who presented with extensive oropharyngeal candidiasis. This effect was independent of the number of cycles and of the previous cycle variation in CD4+ T-cell counts. Finally, mean pre-treatment and retreatment values were not significantly different. The changes in CD4+ T-cell counts in the eight patients with a value of <300/mm^3^ at least once during follow up are presented in Fig. 5. The relationship between adverse events and lymphocyte phenotyping will be studied in a separate report, and therefore will not be presented in the present article.

### Clinical response and CD4+ T-cell changes observed during cycle 2, in cycle 1 non-responders

**Fig. 6:**
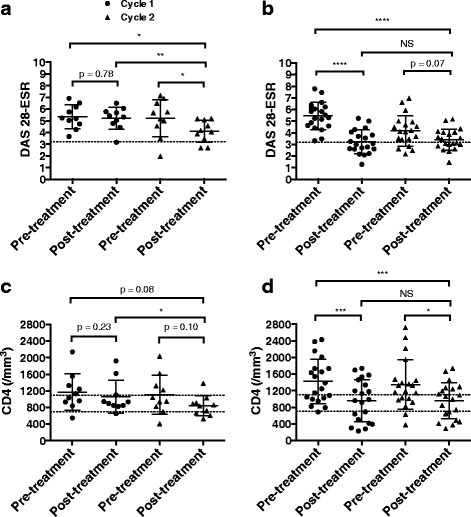
Changes in Disease Activity Score in 28 joints-erythrocyte sedimentation rate (DAS28-ESR) (**a**, **b**) and CD4+ T-cell counts (**c**, **d**) over cycle 1 and cycle 2 in first-cycle non-responders (**a**, **c**) (n = 10) and in first-cycle responders (**b**, **d**) (n = 20). Pre-treatment and 3 or 6 months post-treatment data are shown as scatter dot plots (first cycle non-responders n = 10; first-cycle responders, n = 20). Each *dot* represents one patient and *lines* represent the mean with SD. For DAS28-ESR analysis, the level defining low disease activity is represented (DAS28 ≤ 3.2). Comparison of pre-treatment and post-treatment values was made using Wilcoxon’s matched-pairs signed rank test: ****p < 0.0001, ***p < 0.001, **p < 0.005, *p < 0.05; *NS* not significant

We then focused on 10 patients who were retreated despite a poor response in cycle 1. The DAS28-ESR and CD4+ T-cell changes in these patients are shown in Fig. 6a and c and those in initial responders in Fig. 6b and d. Of the 10 responders, 8 initial non-responders became responders after the second cycle. The cycle-2 post-treatment DAS28-ESR was decreased in these patients compared to cycle 2 pre-treatment and to cycle 1 pre-treatment and post treatment. In initial responders, the post-treatment DAS28-ESR was similar in cycles 1 and 2 and CD4+ T-cell counts were significantly and similarly decreased in both cycles. Conversely, in initial non-responders, CD4+ T-cell counts remained the same in cycle 1; however, in cycle 2 the post-treatment CD4+ T-cell counts tended to be lower than the pre-treatment values in both cycle 1 and cycle 2 (*p* = 0.08 and *p* = 0.10, respectively). Moreover, they were significantly decreased compared to cycle-1 post-treatment values, further suggesting a potential relationship between disease activity and CD4+ T-cell counts in patients with RA receiving RTX.

## Discussion

Repeated cycles of RTX are known to induce sustained and sometimes improved efficacy in patients with RA [14]. We show here that improved clinical response observed in patients who received RTX on an as-needed basis, was associated with repeated variation in CD4+ T-cell counts occurring over successive cycles.

The first finding of this study was that the mean CD4+ T-cell counts were above the upper limit of the reference range at baseline and before each RTX cycle. This result indicates that the homeostasis of CD4+ T cells was disrupted before RTX treatment and when the patients stopped responding. Interestingly, baseline CD4+ T-cell counts were significantly higher in responders than in non-responders on the one hand, and in patients without RF and in those without radiologic evidence of erosion on the other hand. These results might suggest that patients with less severe forms of the disease are more likely to respond to RTX. However, until now only the presence of RF [8, 9] or ACPA [8] has been identified as predictor of clinical response. Larger studies are required to confirm this conclusion and, eventually, to define a predictive threshold of CD4+ T-cell counts value for clinical response.

We have previously reported a substantial decrease in CD4+ T-cell counts in a large majority of patients with RA receiving a first cycle of RTX. A decrease in CD4+ T cells was not observed in the absence of response following the first cycle [11]. The present study shows that a decrease in CD4+ T-cell counts occurred repeatedly along with clinical response in patients who received up to seven cycles of RTX. A slightly additive effect of repeated treatment was seen, with the mean post-treatment CD4+ T-cell count gradually reaching the lower limit of the normal range. Additionally, we found that a second cycle of RTX in initial non-responders led to a clinical response in 8 of 10 patients. The improvement in clinical response following this second cycle was associated with a decrease in CD4+ T-cell counts, which was not observed in the first cycle.

It is known that post-RTX B-cell recovery is a long process [3–7, 15]. Therefore, patients are usually retreated before complete recovery of the B-cell pool. There was a tendency toward a decrease in pre-treatment CD19+ B-cell counts with repeated cycles. This may be due to a biologic effect of RTX, but it may also be explained by the gradual reduction in the time interval between each cycle that we observed. By contrast, an RTX-induced CD4+ T-cell decrease was followed by complete recovery of the circulating CD4+ T-cell pool at the end of each cycle. The time to CD4+ T cell recovery may vary across patients and may relate to the time to relapse. Also we previously reported that there was a greater decrease in the CD4+ T-cell count in patients whose time to retreatment was >12 months than in those whose time to retreatment was <12 months [11]. The assumption that CD4 could be a valuable marker is based on the coincidence between the clinician’s decision whether to retreat and the CD4 recovery. It is indeed very apparent that recovery of CD4+ cells occurs at the time of the relapse. Taken together, these results show that in patients with RA receiving RTX, disease activity is more closely related to CD4+ T-cell variations than to B-cell variations, suggesting that monitoring CD4+ T cells might be more relevant for predicting disease relapse. The present results reopen the issue of re-treatment timing, and suggest that a scheme of RTX administration based on CD4+ T-cell counts might anticipate and eventually avoid disease relapse.

## Conclusions

Repeated post-treatment decrease in CD4+ T-cell counts over successive cycles of RTX occurs in patients with RA. Changes in CD4+ T-cell counts could be a relevant biomarker of RA activity in RTX-treated patients and hence aid the determination of an appropriate treatment schedule. The accuracy of CD4+ T-cell counts as a biomarker of disease activity and/or RTX efficacy needs to be confirmed by further prospective studies.

## Abbreviations

ACPAs: anti-citrullinated protein antibodies
DAS28: Disease Activity Score in 28 joints
ESR: erythrocyte sedimentation rate
EULAR: European League Against Rheumatism
RA: rheumatoid arthritis
RF: rheumatoid factor
RTX: rituximab
